# Cognitive flexibility in urban yellow mongooses, *Cynictis penicillata*

**DOI:** 10.1007/s10071-024-01839-9

**Published:** 2024-03-02

**Authors:** Mijke Müller, Neville Pillay

**Affiliations:** School of Animal, Plant and Environmental Sciences, University of the Witwatersrand, Johannesburg, South Africa

**Keywords:** Attention, Flexibility, Reversal learning, Urban, Yellow mongoose

## Abstract

Cognitive flexibility enables animals to alter their behaviour and respond appropriately to environmental changes. Such flexibility is important in urban settings where environmental changes occur rapidly and continually. We studied whether free-living, urban-dwelling yellow mongooses, *Cynictis penicillata*, in South Africa, are cognitively flexible in reversal learning and attention task experiments (n = 10). Reversal learning was conducted using two puzzle boxes that were distinct visually and spatially, each containing a preferred or non-preferred food type. Once mongooses learned which box contained the preferred food type, the food types were reversed. The mongooses successfully unlearned their previously learned response in favour of learning a new response, possibly through a win-stay, lose-shift strategy. Attention task experiments were conducted using one puzzle box surrounded by zero, one, two or three objects, introducing various levels of distraction while solving the task. The mongooses were distracted by two and three distractions but were able to solve the task despite the distractions by splitting their attention between the puzzle box task and remaining vigilant. However, those exposed to human residents more often were more vigilant. We provide the first evidence of cognitive flexibility in urban yellow mongooses, which enables them to modify their behaviour to urban environments.

**Supplementary Information:**

The online version contains supplementary material available at 10.1007/s10071-024-01839-9.

## Introduction

Cognitive flexibility describes an animal’s ability to continually learn and alter goal-directed behaviour, even as the learning contingencies change (Klanker et al. 2013; Tello-Ramos et al. 2019). Cognitive flexibility enables animals to adapt to rapidly changing environments (Sol 2009), particularly in generalist species that frequently respond to environmental variability (Mettke-Hofmann 2014; Sol et al. 2016). However, not all species have comparable levels of cognitive flexibility, likely as a result of differences in memory retention abilities, with those retaining learned information being less flexible than those with decreasing memory retention over time (e.g. Freas et al. 2012; Tello-Ramos et al. 2018, 2019). Cognitive flexibility and retention trade-off, as well as interspecific variation in levels of cognitive flexibility (Lefebvre et al. 2004), might indicate costs associated with being cognitively flexible (Tello-Ramos et al. 2018). This means that different environments favour either high levels of cognitive flexibility or greater retention (Tello-Ramos et al. 2018). Thus, cognitive flexibility should be retained in some species in rapidly changing environments (Tello-Ramos et al. 2018) as an adaptive response to urban environments (Sol et al. 2013). Our study assesses the occurrence of cognitive flexibility in a small urban carnivore.

Reversal learning is assessed by training an animal to distinguish between two stimuli, typically using a food incentive. Once the animal successfully discriminates between the stimuli, the outcome of the reward is reversed, and the animal must inhibit the previously learned response to obtain the reward (Lucon-Xiccato and Bisazza 2014). Solving such a task involves associative learning followed by reversal learning. Associative learning is considered a conserved form of learning (Papini 2002; Ginsburg and Jablonka 2010; Bennett 2021). Conversely, reversal learning involves unlearning the associative learning component and replacing it with a new association, which requires individuals to behave flexibly. However, solving a reversal learning task is more complex than the initial association (e.g. Buechel et al., 2017).

Animals may make use of various rule-based strategies when solving a reversal learning task, such as (1) making the correct choice and then continuing to make the same correct choice (win-stay strategy), (2) making the correct choice and then changing to the incorrect choice (win-shift strategy), (3) making the incorrect choice and then changing to the correct choice (lose-shift strategy), and (4) making the incorrect choice and then continuing to make the same incorrect choice (lose-stay strategy) in the subsequent trial. Reversal learning requires a win-stay, lose-shift strategy sequence (Nowak and Sigmund 1993; Buechel et al. 2017). In other words, if the outcome of any given stimulus is a reward, an individual should stay and interact with the stimulus to be rewarded consistently. If, however, the outcome is no longer rewarded, the individual should move to the alternative to increase its chances of being rewarded. However, even in the most successful case, this strategy would require an animal to make at least one error immediately following the reversal (Nowak and Sigmund 1993; Buechel et al. 2017).

Performance in reversal learning may be negatively correlated with performance on the previous associative learning (Bebus et al. 2016). Thus, there is a trade-off between initial associative learning and later reversal learning task performance (Bebus et al. 2016). This is related to memory differences; individuals with better memories are likely to be less flexible due to remembering previously learned information at the expense of forgetting more recently learned information (proactive interference), whereas those that are more forgetful may be more flexible since previously learned information are forgotten and instead they remember recently learned information (retroactive interference; Croston et al. 2016). Proactive interference is considered a lower-order process since it occurs involuntarily and represent a lower level of flexibility when compared to rule-based strategies (e.g. win-stay, lose-shift; Mackintosh et al. 1968; Parker et al. 2012; Liu et al. 2016). Similarly, a trade-off between the speed and accuracy might results in slower animals having greater accuracy when solving tasks, and those that solve tasks quicker making more mistakes (e.g. Mazza et al. 2018).

Various species show the ability to inhibit their previously rewarded response through reversal learning, including reptiles (e.g. *Anolis evermanni*, Leal and Powell 2012), fish (e.g. guppies, Lucon-Xiccato and Bisazza 2014), amphibians (poison frog, *Dendrobates auratus*, Liu et al. 2016), and mammals (e.g. raccoons, *Procyon lotor*; striped skunks, *Mephitis mephitis*; coyotes, *Canis latrans*, Stanton et al. 2021). The ability to reversal learn has been attributed to various intrinsic factors (e.g. brain size, Buechel et al. 2017; memory, Croston et al. 2016), but several social and ecological factors may further influence an animal’s level of flexibility, and thus, reversal learning abilities (e.g. sociality, Bond et al. 2007; group-living, Buechel et al. 2017; environmental variability, Tebbich and Teschke 2014; environmental harshness, Croston et al. 2016; seasonality, Rochais et al. 2021). This indicates that cognitive flexibility may be influenced by the immediate environment in which individuals occur, even within the same species or population. Additionally, urbanisation (Federspiel et al. 2017) and food availability (Tebbich and Teschke 2014) predict whether reversal learning will be favoured.

Ideally, animals would be able to interact with and solve tasks without any distractions or interference. In reality, animals must pay as much, if not more, attention to their surroundings in an attempt to detect predators and avoid other dangers, thus dividing their attention among many different tasks. This, however, is cognitively demanding (Griffiths et al. 2004; Zentall 2005). Urban animals are exposed to numerous distractions, such as the presence of humans, vehicular traffic and noise, to which they must adapt (Sol et al. 2013). Thus, being cognitively flexible to switch between multiple cognitively demanding tasks may further aid their success in urban areas.

Attention refers to the selective way in which animals allocate their attention by processing certain stimuli and disregarding others. Selective attention is an animal’s ability to focus on one task, process relevant information, and filter out irrelevant distractions to process and respond to the focal stimulus more efficiently (Zentall 2005; Carlson et al. 2018). This avoids cognitive overload or inappropriate behavioural responses while engaging in a task (Commodari 2017). There are, however, costs with the use of selective attention, such as reduced predator detection rates (Dukas and Kamil 2000). Conversely, divided attention is when an individual focuses on multiple tasks simultaneously and divides its attention equally among the tasks (Parasuraman 1998). Even though this type of attention is ideal to solve tasks in the face of distractions, a possible shortfall of dividing attention is not processing information efficiently due to the high cognitive demand, resulting in a decline in performance (Dukas and Kamil 2001; Griffiths et al. 2004; Zentall 2005).

An alternative mechanism of focusing on multiple stimuli is alternating attention, which involves rapidly shifting attention from one task to another, instead of focusing on the tasks in parallel (Commodari 2017). This allows animals to terminate focus on one task in favour of focusing on a second task, before terminating focus on the second task in favour of focusing again on the first (Commodari 2017). This rapid shifting of attention is attributed to the inability of animals to focus on two tasks simultaneously (Parasuraman 1998), while enabling animals to respond to stimuli in their environment as they appear. Similar to reversal learning, the dividing or shifting of attention to focus on multiple stimuli may indicate cognitive flexibility, which involves switching between tasks as their priorities change (Diamond 2013).

Cognitive flexibility is closely linked with two aspects of memory: short-term memory and working memory. The use of rule-based strategies to solve reversal learning tasks (i.e. win-stay, lose-shift) requires adequate short-term memory since the individual has to remember which stimulus was previously rewarded (Buechel et al., 2017), and the success of each trial will depend on the animal’s memory of the previous trial (Gonzalez et al. 1967; Kamil, 1985; Buechel et al. 2017). Thus, cognitive flexibility may not exclusively depend on the ability to learn but may be coupled with the ability to retain newly acquired information (Gonzalez et al. 1967; Croston et al. 2016). Working memory has a limited capacity and may be further important in reducing proactive interference (Diamond 2013). Limitations in working memory may result in processing deficits and reduced performance during divided attention, or inhibit divided attention altogether. As a result, the ability to divide attention increases with reduced task similarity (McLeod 1977), more practice (Spelke et al. 1976) and reduced task difficulty (Sullivan 1976).

Our study species is the yellow mongoose, *Cynictis penicillata*, a small (±300 mm; 500–1000g; Kingdon et al. 2013), generalist carnivore (Bizani 2014) distributed throughout southern Africa (Do Linh San et al. 2015). It is adapted to urban environments where it often ingests anthropogenic food items (Cronk and Pillay 2018, 2019a, b). In a previous study, we established that yellow mongooses in an urban location could solve a novel task of increasing complexity (Müller and Pillay 2023). Since mongooses are able to learn to solve a novel problem in a rapidly changing urban environment, we expected that they would have some level of cognitive flexibility, allowing them to alter their behaviour based on changes in their environment.

We studied the learning capabilities of yellow mongooses to assess their cognitive flexibility in reversal learning and attention task experiments, both of which had not previously been tested in yellow mongooses. We hypothesised that the urban yellow mongooses would exhibit evidence of cognitive flexibility by learning to inhibit a previously learned response (reversal learning), as well as solving a task in the presence of distraction (attention task). We predicted that after an associative learning phase, the mongoose would initially increase the latency to consume the preferred food item directly following the reversal (since at least one error is expected directly following a reversal; Mackintosh et al. 1968) and that the latency to consume would decrease over the successive trials. Similarly, we expected a decrease in the success rate directly following the reversal with an increase over the successive trials as mongooses would at first behave according to the initial association but successfully relearn which box contained the preferred food item. For the attention task experiments, we assessed whether yellow mongooses divided their attention between two tasks by successfully solving a task in the face of different levels of distraction. We predicted that mongooses would split their attention between the task and vigilance behaviour and solve the puzzle box task at every level of distraction. Since our study population was located in an urban environment at different distances from people, we assessed whether the level of human contact was associated with the mongooses’ cognitive flexibility. We expected that their reversal learning ability and attention would not differ between two locations with different levels of human presence or with the proximity to human residents since we previously showed that problem-solving abilities did not differ between the two study locations (Müller and Pillay 2023).

## Materials and methods

### Study area

This study took place from April to December 2020 at two locations in the urbanised Meyersdal area, Johannesburg, South Africa. The Meyersdal Nature Estate (26°18′ 08.2″ S 28°04′ 55.9″ E; 300 ha) consisted of a residential area and a separate, fenced-off nature area where the yellow mongoose colonies were located. The Meyersdal Eco Estate (26°17′ 03.6″ S 28°04′ 50.8″ E; 480 ha) had no fenced-off nature area, and the mongoose colonies were dispersed throughout the residential area. The two locations were separated by a double-laned tar road that the yellow mongooses rarely crossed (Cronk and Pillay 2021). A total of ten sites (colonies) were selected for this study, five in the Nature Estate and five in the Eco Estate. These were selected because of regular occurrence of yellow mongooses and a distance of at least 200 m from any other site to ensure independent colonies were selected (urban yellow mongooses’ home ranges are approximately 0.13 km^2^ on average; Cronk and Pillay 2019a, 2021).

### Experimental design and protocol

Clear Perspex puzzle boxes were used for both experiments (Fig. S1; Supplementary Material). The puzzle boxes consisted of a lid that could be opened and closed via hinges. The boxes also had small holes along the sides and top to allow mongooses to detect food incentives placed within (1 teaspoon meat offcuts/bread) via olfactory cues (in addition to the visual cues through the clear box). The mongooses were trained to open the puzzle boxes at all sites (Müller and Pillay 2023). This included two stages: stage 1, where the mongooses were exposed to a puzzle box with an open lid allowing for habituation; and stage 2, where mongooses were exposed to a puzzle box with a mostly closed lid propped up with a stick, leaving a 1 cm opening. The mongooses had to obtain the food incentive from the puzzle box successfully for a total of five trials at stage 1 before proceeding to stage 2. After the mongooses successfully obtained the food incentive from the puzzle box at stage 2 for a total of five trials, the lids of the puzzle boxes were closed entirely, and the experiments commenced.

#### Individual identification

Yellow mongooses are predominantly solitary foragers (le Roux et al. 2008; Manser et al. 2014), and as a result, the same focal individual consistently visited the experiments and continually engaged with the puzzle boxes at each site. This was confirmed using facial recognition software developed with the assistance of the School of Computer Science and Applied Mathematics at the University of the Witwatersrand. This software used facial markers and machine learning to identify individual mongooses from high quality photographs obtained from our camera trap footage (likelihood models showed a high facial recognition accuracy of 90.2% with a confidence range of 88.1% to 97.3%; unpublished data). Only adult mongooses participated in this study, and juveniles never attempted to solve the puzzle box task. Males and females could not be distinguished due to yellow mongooses’ lack of sexual dimorphism, and therefore sex was not considered in this study. Furthermore, historical trapping events in the Meyersdal area revealed that yellow mongooses are extremely trap-shy and never again engaged with trapping cages once trapped and collared (Cronk, personal communication). As a result, permission for additional trapping and collaring of individuals was not obtained from estate management, especially during the COVID-19 pandemic when veterinary assistance was limited.

#### Reversal learning experiment

For the reversal learning experiment, mongooses underwent two learning phases: an associative learning phase and a reversal learning phase. During the associative learning phase, mongooses were trained to associate a specific puzzle box with either a preferred or non-preferred food incentive. Two puzzle boxes with closed lids were placed next to each other (the width of one puzzle box apart). Two differently shaped objects (a small plastic animal figurine and a pebble of a similar size obtained from the immediate environment) were attached to the lid of each box, respectively, providing the mongooses with a distinct visual difference between the two boxes. The boxes remained in the same position throughout this study, allowing the mongooses to use their position in the environment as an additional cue (Figure S2a; Supplementary Material). Two different food incentives were used: a preferred food item and a non-preferred food item. Consistent with Cronk and Pillay (2018), a pilot study conducted prior to this experiment revealed that the mongooses preferred meat which was selected as the preferred food item. However, in contrast to Cronk and Pillay (2018) who found that bread was the second-most preferred food item, we found that the mongooses rarely consumed bread, which was selected as the non-preferred food. The preferred food was placed in the first box (with the figurine), and the non-preferred food item in the second box. This was kept consistent for the duration of this associative learning phase. To ensure that the mongooses did not choose the puzzle box based on olfactory cues, the holes in the puzzle boxes were covered for the duration of this experiment, and both boxes were exposed to the scent of both food items before each trial by placing the opposite food type in each puzzle box before replenishing the boxes with the correct food type.

The experiment was set up between 6:00 and 12:00 daily when yellow mongooses were the most active (Cronk and Pillay 2019a, 2020). After the initial set-up at one site was completed, the same experiment was set up at the other sites (i.e. the experiment was conducted at all sites simultaneously). We visited each site twice daily (between 6:00 and 12:00) and the puzzle boxes were reset if the mongooses had consumed the food during the previous trial. This allowed for a maximum of two trials per site per day while minimising the time between trials. Resetting the experiment involved clearing out any remaining food from the puzzle boxes and replenishing the boxes with the same fresh food incentives. The same puzzle boxes were used for the duration of the experiment at each site (unless the puzzle box was destroyed by large herbivores or removed by human residents, in which case it was replaced with a new puzzle box).

Browning^®^ Trail Cameras (Model BTC-8A; 55° field of view; Browning 2019) triggered with a motion sensor were set up between 1 and 2 metres away from the experiment with an unobstructed view of the puzzle boxes. Each trial started as soon as a mongoose entered the camera’s field of view (1–2 m across) and ended as soon as the mongoose consumed the preferred food item. From the video footage, the box contacted, opened and the food consumed first was recorded. Individual trials were recorded as successful (box containing the preferred food item was contacted first, opened first, and food consumed first), successful with error (box containing non-preferred food item was contacted first, whereafter the mongoose immediately opened the box containing the preferred food item and consumed the preferred food item), or unsuccessful (box containing non-preferred food item was opened before box containing preferred food item). From the camera trap footage, the number of successful, successful with error, and unsuccessful trials were recorded. The latency to consume the preferred food reward was recorded for each trial as the number of seconds elapsed from the moment the mongoose entered the camera’s view until it consumed the preferred food item. The box contacted and opened first, and the food item consumed was recorded. Mongooses had the opportunity to visit both boxes during every trial, even if they selected the box with the non-preferred food item first. Trials that were successful with error were considered successful since the mongooses were able to correct their behaviour after contacting the incorrect box initially. The learning criterion consisted of 7 successful trials out of 10 consecutive trials. Once this criterion was reached, the associative learning phase was terminated, and the reversal learning phase commenced.

During the reversal learning phase, the food items in the boxes were reversed; the box previously containing the preferred food now contained the non-preferred food, and vice versa (Figure S2b; Supplementary Material). The same criterion as the previous phase was used (7 successful trials out of 10 consecutive trials). Once again, the number of successful, successful with error, and unsuccessful trials, the latency to consume the preferred food, the box visited first, and the food item consumed first was recorded. The straight-line distance between each site and the nearest human residence was measured using Google Earth^™^ and used as a measure of the effect of human residence proximity on the reversal learning abilities of yellow mongooses.

In order for this experiment to truly test mongooses’ reversal learning abilities, we needed mongooses to associate a learned cue (visual and spatial) with a particular food incentive. If mongooses were able to smell the food, they would not have had to learn any associations and could instead directly approach the box containing the preferred food type. In this case, their behaviour would be based on instantaneous decision-making without any learning occurring. A control test, similar to the one by Lucon-Xiccato and Bisazza (2014), was performed after the reversal learning experiment at eight of the ten sites in order to validate the experimental design that mongooses were using visual cues to select a puzzle box rather than olfactory cues to determine the box with the preferred food. In this control test, the same experiment was done, but the puzzle boxes had no object, and their position/orientation was randomly set (Figure S2c; Supplementary Material). Therefore, the visual and spatial cues of the boxes were not obvious. From this test, we found that the mongooses did not select the box containing the preferred food incentive more often than predicted by chance, and instead contacted the box containing the non-preferred food item in the absence of the visual and spatial cues previously provided (75% of all control trials). This indicates a lack of use of olfactory cues in decision-making.

#### Attention task experiment

The attention task experiments were conducted directly following the reversal learning experiments at the same sites (the same focal individuals participated in all trials during both experiments as confirmed using facial recognition technology, see ‘Individual identification’). For the attention task experiment, mongooses were provided with a single puzzle box with the lid closed and containing meat as the food incentive (no stage 1 and stage 2 training was required since mongooses already knew how to open the puzzle box). Zero, one, two or three distraction objects were placed around the box, providing the mongooses with varying levels of distraction. The distraction objects consisted of a stick (approximately 20 cm in length) nailed to the ground, with shiny silver strips of plastic attached to the stick. The strips moved with the wind, creating unpredictable distractions while the mongooses engaged with the puzzle box (Figure S3; Supplementary Material). The position in which the distraction objects were placed around the box and the number of objects were changed randomly for each trial to avoid the mongooses habituating to the objects. Each level of distraction was repeated for a total of three trials per distraction level at each site (i.e. a total of 12 trials per site). This experiment was set up at all sites daily and revisited once per day to replenish the food incentive (this allowed for a maximum of two trials per site per day).

From camera trap footage, the latency to consume the food incentive was recorded for every trial to ascertain whether the mongooses were able to efficiently solve the puzzle box task while experiencing distractions. Additionally, we recorded the frequency of vigilance behaviour (defined as the ceasing of all activity, alert, scanning the environment and standing upright on hind legs). Finally, we assessed whether the proximity of human residences was associated with the ability to solve tasks.

### Data analyses

All statistical analyses were conducted using R Statistical Software (R version 3.4.3; R Core Team 2013). Shapiro-Wilk tests were used to test for normality. Non-normal variables were transformed using a Box-Cox transformation (R package: MASS), and if variables did not normalise after transformation, non-parametric tests were used. Parametric tests used for normal and transformed variables included linear mixed effect models (LMER) and non-parametric tests used for non-normal variables that could not be transformed include generalised linear mixed effect models (GLMM) and Spearman’s rank correlation. For the reversal learning experiment, an LMER (R packages: lme4, lmerTest) was used to analyse differences in the latency to consume the preferred food item by location and treatment (learning phase). The two treatments were a repeated measures design, with location and treatment as fixed effects, trial and site as random factors and the proximity to human residents as a covariate. A Tukey post hoc test was used to determine pairwise differences for significant fixed factors (general linear hypothesis; R package: multcomp). A linear regression was used to assess whether the latency to consume the preferred food item changed over the trials. A GLMM (family = binomial; link = logit) was used to tests for any trade-offs (1. speed-versus-accuracy and 2. associative learning versus reversal learning) by assessing whether 1. the mean latency to consume the food and 2. treatment (learning phase) predicted individual accuracy (measured as success rate) at each learning phase. Location, treatment (learning phase) and the latency to consume the correct food were fixed effects and trial and site (individual mongoose) were random factors. The success rate was calculated as a percentage of the number of successful trials out of the total number of trials per site for each learning phase. Spearman’s rank correlation tests were performed post-hoc. Finally, a Spearman’s rank correlation was used to assess whether the success rate changed over the trials.

For the attention task experiment, a GLMM was used to analyse differences in the (i) latency to contact the puzzle box (family = Gamma; link = log) and (ii) vigilance frequency (family = Poisson; link = log), and an LMER to analyse the difference in (iii) latency to consume the food incentive with the different levels of distraction at the two locations. The various treatments (number of distraction objects) were a repeated measures design, with location, and treatment as fixed effects, trial and site as random factors and the proximity to human residents as a covariate. The interaction between treatment and location was included as a fixed effect in the first two models only. A Tukey post hoc test was used to determine pairwise differences for significant fixed factors (general linear hypothesis; R package: multcomp). A Spearman’s rank correlation was used to analyse whether the latency to consume the food incentive was significantly correlated with the vigilance frequency.

We used AIC, R^2^ and f^2^ values to calculate the effect sizes of models including and excluding interactions between fixed effects. Models with smaller AIC values were considered the most appropriate. In addition, for models with f^2^ scores that indicated small effect sizes for interaction effects (< 2%), we considered only first order analyses.

## Results

A total of 10 individual mongooses participated in this study, five from the Nature Estate and five from the Eco Estate.

### Reversal learning experiment

#### Latency to consume

**Fig. 1:**
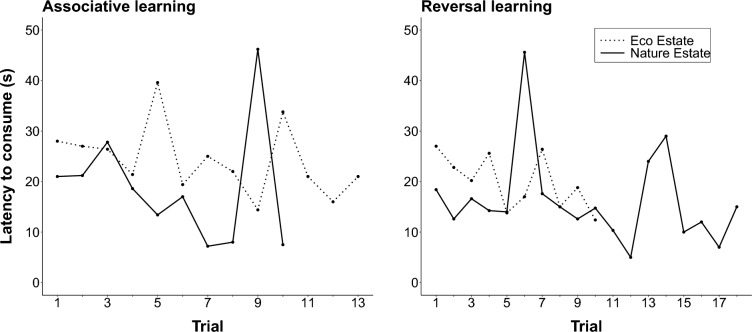
The mean latency to consume (s) the preferred food item by yellow mongooses in the Meyersdal Eco Estate and Meyersdal Nature Estate during the associative and reversal learning phases of a reversal learning experiment

Location (χ^2^ = 2.23, df = 1, p = 0.135; LMER), learning phase (associative versus reversal; (χ^2^ = 0.71, df = 1, p = 0.399) and the proximity to the nearest human residents (χ^2^ = 0.001, df = 1, p = 0.972) were not significant predictors of the latency to consume. We predicted that the latency to consume the preferred food type would be longer during the first trial of each phase and decrease during subsequent trials as the mongooses learned which box contained the preferred food type. In each location, the mean latency to consume the food for all sites generally decreased over the trials and during each treatment (Fig. 1). During the associative learning phase, the latency to consume the food was the highest in the Eco Estate compared to the Nature Estate during the first and last trials, but decreased to below the initial latency for the final trial in each location (Nature Estate: $\hat {\upbeta}$ = − 0.46, adjusted R^2^ = − 0.11, p = 0.745; Eco Estate: $\hat {\upbeta}$ = − 0.71, adjusted R^2^ = 0.08, p = 0.177). During the reversal learning phase, the latency to consume the food was initially similar to the first trial of the associative learning phase, but longer than the final trial of the associative learning phase. The latency to consume decreased more rapidly in the Eco Estate (area of more human contact) than in the Nature Estate (area of reduced human contact) and was lower during the final trial in the Eco Estate than in the Nature Estate (Nature Estate: $\hat {\upbeta}$ = − 35, adjusted R^2^ = − 0.02, p = 0.418; Eco Estate: $\hat {\upbeta}$ = − 1.09, adjusted R^2^ = 0.30, p = 0.060). The latency to consume the food during the final trial of the reversal learning stage in the Nature Estate was lower than it was during the initial trial in the same location. However, from trial 13 onwards, the latency to consume in this location was affected by one site only where the success criterion was not reached. While the trendlines were not significant, the latency to consume decreased generally over successive trials in both locations and during both treatments, as indicated by the negative $\hat {\upbeta}$-values.

#### Success counts and success rates

**Fig. 2:**
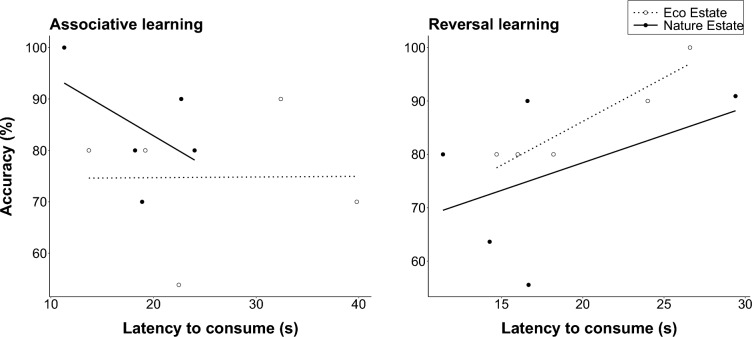
The relationship between the accuracy and latency of solving a puzzle box task by yellow mongooses in the Meyersdal Eco Estate and Meyersdal Nature Estate during the associative learning and reversal learning phases during a reversal learning experiment. The trendline is significant for the Eco Estate during the reversal learning phase (Spearman rank test)

The latency to consume the preferred food item (χ^2^ = 24.28, df = 1, p < 0.001) was the only predictor of success, whereas location (χ^2^ = 0.37, df = 1, p = 0.541) and learning phase (χ^2^ = 0.62, df = 1, p = 0.432) were not. There was a significant, strong, positive correlation between latency to consume the preferred food and accuracy during reversal learning in the Eco Estate, so that accuracy increased with increased time taken to consume the preferred food (r_s_ = 0.89, p = 0.041; Fig. 2). There were no such correlations between the latency to consume the preferred food and accuracy during the associative learning phase in either location (Eco Estate: r_s_ = − 0.15, p = 0.805; Nature Estate: r_s_ = − 0.36, p =0.553) or during the reversal learning phase in the Nature Estate (r_s_ = 0.30, p = 0.683).

**Fig. 3:**
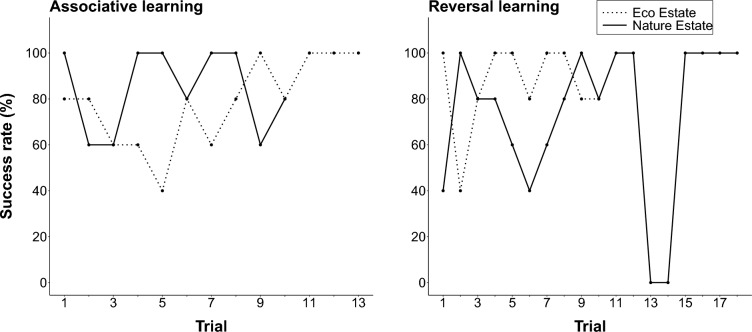
The percentage success by yellow mongooses in the Meyersdal Eco Estate and Meyersdal Nature Estate for each trial during the associative learning and reversal learning phases during a reversal learning experiment

To investigate the patterns of success over the various trials, we calculated the success rate using the proportion of successful trials at each location (Fig. 3). The success rate differed between the two locations and over the various trials. During the associative learning phase, mongooses reached the 70% success rate criterion at ten trials in the Nature Estate and 13 trials in the Eco Estate. Mongooses in the Nature Estate had a 100% success rate at all sites during the first trial, which dropped to 60% during trials 2 and 3, and fluctuated between 60% and 100% for the remainder of the trials (r_s_ = − 0.03, p = 0.928). In the Eco Estate, mongooses initially had an 80% success rate which decreased to 40% over the first five trials. Thereafter, their success rate increased until they reached 100% success at trial 11 and maintained this success rate for the remainder of the trials (r_s_ = 0.67, p = 0.012). During the reversal learning phase, the mongooses reached the 70% success rate criterion at 18 trials in the Nature Estate and ten trials in the Eco Estate. In the Nature Estate, the mongooses’ success rate dropped from 80% during the last trial of the associative learning phase to 40% during the first trial of the reversal learning phase. Thereafter, the success rate fluctuated between 40% and 100% until they reached 100% at all sites during consecutive trials at trials 11–12 and again at trials 15–18 (r_s_ = 0.37, p = 0.133). In the Eco Estate, mongooses maintained their 100% success rate from the last trial of the associative learning phase during the first trial of the reversal learning phase. During trial 2, the success rate dropped to 40%, whereafter it fluctuated between 80% and 100% for the remainder of the trials (r_s_ = − 0.03, p = 0.927).

### Attention task experiment

#### Latency to contact

**Fig. 4:**
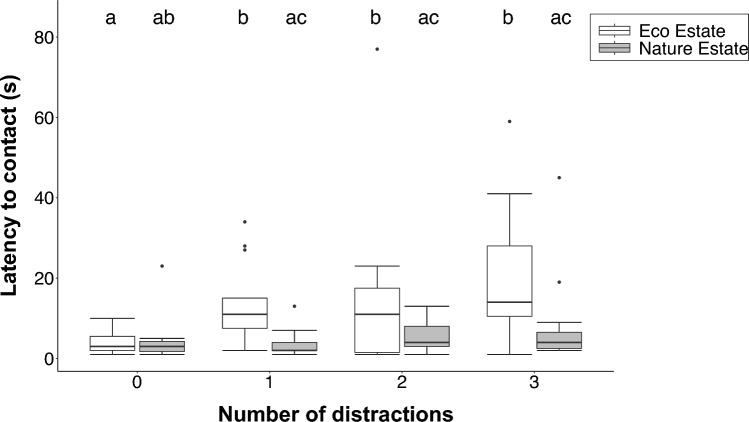
The latency to consume food (s) by yellow mongooses in the Meyersdal Eco Estate and Meyersdal Nature Estate in the presence of zero, one, two and three distractions. The boxes indicate the upper and lower quartiles, the horizontal lines indicate medians and the error bars indicate confidence intervals. Significant differences are shown by different alphabet letters

The number of distractions (χ^2^ = 28.99, df = 3, p < 0.001; GLMM) and the interaction between the location and the number of distractions (χ^2^ = 12.45, df = 3, p = 0.006) were significant predictors of the latency to contact the puzzle box, whereas location (χ^2^ = 0.08, df = 1, p = 0.775) and the proximity to the nearest human residents (χ^2^ = 0.81, df = 3, p = 0.369) were not. The latency to contact the puzzle box was significantly shorter when there were zero distractions present compared to one (z = 4.07, p < 0.001), two (z = 3.87, p < 0.001) and three (z = 3.97, p < 0.001) distractions. The latency to contact the puzzle box was significantly longer in the Eco Estate than the Nature Estate in the presence of distractions (one distraction: z = 3.95, p < 0.001; two distractions: z = 2.52, p = 0.012; 3 distractions: z = 3.16, p = 0.002). Additionally, the latency to contact the puzzle box was significantly longer at one (z = − 4.07, p < 0.001), two (z = − 3.87, p = 0.001) and three (z = − 3.97, p < 0.001) distractions in the Eco Estate (Fig. 4).

#### Latency to consume

**Fig. 5:**
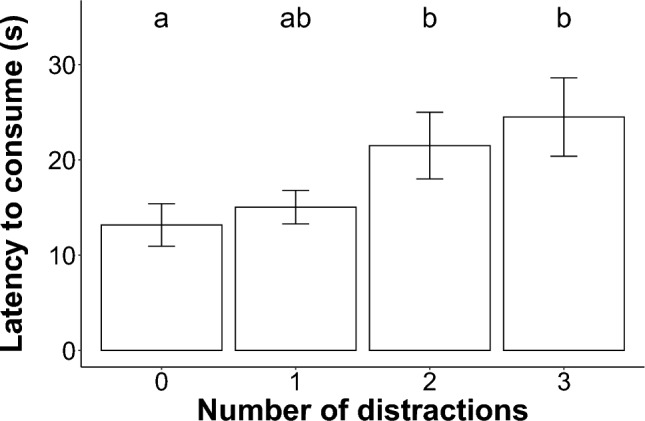
The mean latency to consume (s) the food by yellow mongooses in the presence of zero, one, two and three distractions. Error bars indicate standard error. Significant differences are shown by different alphabet letters

The number of distractions present at the puzzle box was the only significant predictor of the latency to consume the food (χ^2^ = 10.92, df = 3, p = 0.012; LMER; Fig. 4). Location (χ^2^ = 1.20, df = 1, p = 0.273), proximity to human residents (χ^2^ = 0.24, df = 1, p = 0.626) and the interaction between the location and number of distractions (χ^2^ = 6.32, df = 3, p = 0.097) were not significant predictors of the latency to consume the food. The latency to consume the food incentive was significantly longer when two (z = 2.59, p = 0.047) and three (z = 3.08, p = 0.011) distractions were present compared to when zero distractions were present (Fig. 5).

#### Vigilance

**Fig. 6:**
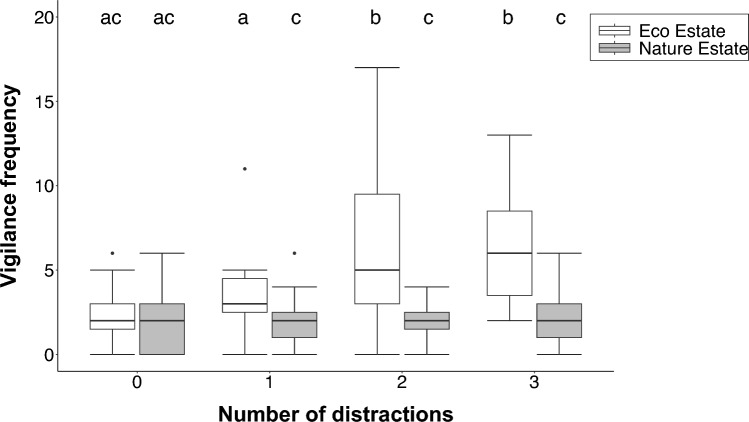
The vigilance frequency by yellow mongooses in the Meyersdal Eco Estate and Meyersdal Nature Estate in the presence of zero, one, two and three distractions. The boxes indicate the upper and lower quartiles, the horizontal lines indicate medians and the error bars indicate confidence intervals. Significant differences are shown by different alphabet letters

The number of distractions (χ^2^ = 33.86, df = 3, p < 0.001; GLMM) and the interaction between the number of distractions and location (χ^2^ = 10.23, df = 3, p = 0.017) were significant predictors of the frequency at which mongooses were vigilant, whereas location (χ^2^ = 1.83, df = 1, p = 0.177) and the proximity to human residents (χ^2^ = 1.48, df = 1, p = 0.224) were not. The mongooses were significantly more vigilant when two or three distractions were present compared to zero (z =4.99, p < 0.001; z = 5.03, p < 0.001) or one (z = 3.09, p = 0.010; z = 3.11, p = 0.010) distraction. The mongooses in the Eco Estate were significantly more vigilant than those in the Nature Estate in the presence of distractions (one distraction: z = 2.50, p = 0.013; two distractions: z = 4.13, p < 0.001; 3 distractions: z = 4.03, p < 0.001). Additionally, the mongooses in the Eco Estate were significantly more vigilant at two and three distractions compared to zero (z = − 4.98, p < 0.001; z = − 5.03, p < 0.001) and one (z = − 3.09, p = 0.011; z = − 3.11, p = 0.010) distraction (Fig. 6).

**Fig. 7:**
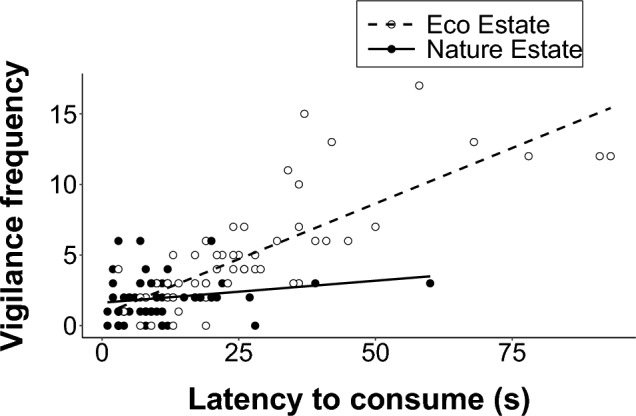
The relationship between the latency to consume (s) the food incentive and the frequency of being vigilant by yellow mongooses in the Meyersdal Nature Estate and the Meyersdal Eco Estate during an attention task experiment. The trendline is significant for both locations (Spearman rank test)

There was a significant, strong positive correlation between the latency to consume (s) and the frequency at which mongooses displayed vigilance behaviour in the Eco Estate (r_s_ = 0.80, p < 0.001), and a significant, weak positive correlation in the Nature Estate (r_s_ = 0.27, p = 0.035). As the vigilance frequency increased, so did the latency to consume (Fig. 7).

## Discussion

Reversal learning experiments were conducted to assess whether a population of urban-dwelling yellow mongooses is cognitively flexible. This was especially of interest since cognitive flexibility is thought to be an important adaptation for urban-living, allowing species to rapidly adjust to environmental variation (Tello-Ramos et al. 2018; Sol et al. 2013). The mongooses in our study were indeed capable of reversal learning, but took a longer time to solve the task during the first trial of the initial associative learning and reversal learning phase compared to the last trial of the respective phases. Similarly, the mongooses’ success rates fluctuated with lower success in earlier trials and improved success during later trials. The mongooses were also able to split their attention between solving the puzzle box task and remaining vigilant to the distraction objects at each level of distraction. However, task solving efficiency declined with increased distraction in the Eco Estate, the location with more human disturbance.

As predicted, during the reversal learning experiment, the mongooses generally took longer to successfully complete the task (consume the preferred food) during the first trial (compared to the final trial) of each learning phase, with a general (non-significant) decrease in consumption latency over time, indicating that they were able to solve the task faster over successive trials. This improvement in solving speed possibly indicates learning in which the mongooses were able to select the correct box quicker by using the visual and spatial cues as associations. The increase in the latency to consume the food during the first trial of the reversal learning phase was related to spending more time locating the correct box after visiting the previously rewarded box initially. However, after they learned that the alternative box contained the preferred food item, the latency decreased again.

We expected a lower success rate directly following the reversal, since animals are likely to make at least one mistake directly following a reversal when using rule-based strategies, after which this probability will decrease (Mackintosh et al. 1968). The mongooses’ success rate was generally the lowest between the first two trials of each learning phase, and then it largely increased or fluctuated between 80% and 100%. In some cases, there was a significant drop in success rate for a single trial, but the success rates were never below 40%. The significant drop in success rate during the reversal learning phase in the Nature Estate was attributed to the fact that a mongoose at only one site required more than 13 trials to reach the success criterion. The mongooses thus had a high success rate overall and the majority appeared to learn the rule of the task within the first few trials as those in the Nature Estate were able to reverse after 4 trials whereas those in the Eco Estate required 9 trials to consistently reverse their response.

Cognitive style, the manner in which an individual approaches a specific problem, often involves trade-offs (Bebus et al. 2016; Mazza et al. 2018). For example, some individuals may be better at learning initial associations, whereas others may be more flexible and perform better at a reversal learning task (e.g. Mazza et al. 2018). Similarly, animals may show a speed-vs-accuracy trade-off, where successfully solving a problem requires more time, and solving a problem quicker can lead to more errors (e.g. Mazza et al. 2018). The mongooses in our study did not show a trade-off between initial acquisition and reversal learning, suggesting that the mongooses did not experience proactive interference, and instead, likely used rule-based strategies for reversal learning success. However, the mongooses did exhibit a speed-vs-accuracy trade-off during the reversal learning phase in the Eco Estate, where slower mongooses were more accurate at solving the task. Slower consumption latencies when solving tasks in the Eco Estate were likely a result of increased vigilance frequencies due to a higher perceived predation risk in this area of increased human activity. However, the higher accuracy may allow mongooses to continually learn to solve tasks, even if they do so at a slower rate. This result brings into question whether the speed of solving a task alone is an accurate representation of cognitive performance, a topic which has been debated at length (e.g., Griffin et al. 2015) but may be particularly important for urban animals which face distractions constantly. Additionally, the lack of a significant relationship between speed and accuracy in this study may further be attributed to the small sample size. While not significant, mongooses in the Nature Estate appeared to be less accurate when solving the task at a slower rate during the associative learning phase but improved their accuracy with slower consumption latencies during the reversal learning phase. However, whether this trend is representative of the population remains to be explored further with a larger sample size.

The mongooses in the Eco Estate (greater human presence) displayed neophobia towards the distractions, whereas those in the Nature Estate (lower human presence) did not show similar neophobia. This result is consistent with our previous findings that yellow mongooses in the Eco Estate are generally more neophobic than those in the Nature Estate (Müller and Pillay 2023). This difference in neophobia, expressed as hesitancy, is typically considered to be a baseline that differs between species and populations, but can decrease over time as an animal becomes used to the novelty (e.g., Greggor, 2016). There was a notable difference in human presence and disturbance between the two locations, likely resulting in more and less neophobic individuals between the locations. Even though urban animals have consistently shown reduced levels of neophobia (e.g., Jarjour et al. 2019), increased neophobia in urban areas may be the result of perceived risk of predation (by the presence of domestic animals, St Clair et al. 2010; increased disturbances generally, Gill 2007) and the differences in neophobia thus likely due to differences in the levels of human disturbance (directly as the result of human-mongoose interaction and indirectly though the presence of domestic animals, noise, vehicular traffic, and so on) between the two locations. Nevertheless, the mongooses solved the puzzle box task at all levels of distraction, indicating that they could engage in cognitively demanding activities despite being surrounded by distractions. However, mongooses were distracted by the artificial distractions when the level of distraction was high (two or three distractions). It appears that mongooses did not utilise selective attention since the distraction level influenced their overall speed at solving the task and resulted in vigilance behaviour, suggesting an inability to filter out the distraction (Zentall 2005; Carlson et al. 2018). Instead, the mongooses more likely divided their attention between the two tasks (solving the task and vigilance), or they were alternating their attention rapidly between the two tasks. To understand their processing of the stimuli, it is necessary to investigate their performance in both behaviours.

The mongooses in the Nature Estate showed no changes in vigilance frequency with an increase in distraction level, whereas those in the Eco Estate were vigilant more frequently with two or three distractions. The correlation between the duration of completing the task and the vigilance frequency showed that the mongooses in the Eco Estate were more vigilant, and also took significantly longer to solve the puzzle box task. If mongooses used divided attention at high levels of distraction, we would have expected worse performance at the puzzle box task, but no change in vigilance frequency and no correlation between the latency to consume and vigilance frequency. This is because divided attention involves the simultaneous processing of both stimuli, which would have resulted in considerably lower vigilance (scored as the termination of all other activities). Instead, the correlation between the vigilance and latency to consume in the Eco Estate concurs with the description of alternating attention, where an individual terminates focus on one task in favour of another, and rapidly shifts their attention back and forth between the two tasks (Commodari 2017). In doing so, the time spent on one task would influence the time spent on another. Similarly, an increase in the number of distractions was expected to increase vigilance frequency and would increase the latency to solve the puzzle box task since the mongooses shifted their attention towards and away from the puzzle box more often. The weak correlation between vigilance frequency and the latency to consume the food in the Nature Estate is more consistent with the definition of divided attention since these individuals performed worse at the puzzle box task with increased distraction (as indicated by the overall latency to consume) but showed no changes in vigilance frequency. A definite conclusion regarding the attentional mechanism utilised by mongooses in this study (divided versus alternating attention) would be implausible without speculation. Additionally, the increased neophobia towards the distraction objects exhibited by mongooses in the Eco Estate may have contributed to their heighted vigilance frequency making it difficult to discern between the true effects of attention allocation and neophobic response. Nevertheless, mongooses in both locations were evidently capable of focusing their attention on two tasks (solving the puzzle box task and remaining vigilant) successfully. This ability to prioritise the processing of stimuli as it becomes relevant in the environment, by focusing on, and switching between, two tasks, suggests cognitive flexibility (Diamond 2013).

Since the ability to divide attention improves with practice (Spelke et al. 1976), it is possible that the mongooses may have used selective attention and divided (or alternating) attention in different circumstances, especially since these two types of attention do not use different brain processes, at least in humans (Hahn et al. 2008). For example, the mongooses may have used divided attention when the level of distraction was intermediate (one distraction) but switched to alternating attention as the distractions increased (two or three distractions), or they may use divided/alternating attention initially but switch to selective attention as it becomes more apparent with enough trials that the distractions pose no threat. In this way, switching between the mechanisms of attention may further indicate cognitive flexibility in urban yellow mongooses, although this was not tested in the present study.

In conclusion, cognitive flexibility is the ability to switch between task rules or shift attention between tasks, adjusting behaviour accordingly (Scott 1962; Diamond 2013). Overall, the mongooses showed evidence of cognitive flexibility by means of two common experimental indicators: the ability to learn and switch between task rules during reversal learning experiments, and the ability to shift attention to relevant environmental cues as their priorities changed during attention task experiments. Not only were they capable of simple associative learning, but they could further learn to solve a reversal learning task. Furthermore, these mongooses were capable of solving a puzzle box task in the face of many distractions, likely through splitting their attention between the tasks, but their task performance declined with more distractions. The results from the reversal learning experiment did not differ between the two study locations, indicating that the level of human disturbance had no effect on their cognitive flexibility. However, the mongooses in the two locations behaved differently when having to divide their attention, perhaps as a result of differences in levels of neophobia. Being cognitively flexible probably enables the mongooses to modify their learned responses to environmental changes and focus their attention on various relevant environmental stimuli simultaneously, which may contribute to their success in urban habitats.

## Supplementary Information

Below is the link to the electronic supplementary material.

Supplementary file1 (PDF 1135 KB)

## Data Availability

The data used for the analyses in this study are available at https://doi.org/10.6084/m9.figshare.23895909.v1.

## References

[CR1] Bebus SE, Small TW, Jones BC, Elderbrock EK, Schoech SJ (2016) Associative learning is inversely related to reversal learning and varies with nestling corticosterone exposure. Anim Behav 111:251–260. 10.1016/j.anbehav.2015.10.027

[CR2] Bennett MS (2021) What behavioral abilities emerged at key milestones in human brain evolution? 13 hypotheses on the 600-million-year phylogenetic history of human intelligence. Front Psychol 12:685853. 10.3389/fpsyg.2021.685853 34393912 10.3389/fpsyg.2021.685853 PMC8358274

[CR3] Bizani M (2014) Diet of the yellow mongoose (*Cynictis penicillata*) in the Albany Thicket Biome of South Africa. Unpublished Master of Science dissertation. University of Fort Hare, pp 1–130

[CR4] Bond AB, Kamil AC, Balda RP (2007) Serial reversal learning and the evolution of behavioral flexibility in three species of North American corvids (*Gymnorhinus cyanocephalus*, *Nucifraga columbiana*, *Aphelocoma californica*). J Comparat Psychol 121(4):372–379. 10.1037/0735-7036.121.4.372 10.1037/0735-7036.121.4.372 18085920

[CR5] Browning. (2019). Browning Trail Cameras: Advantage SpecOps Full HD Video Model BTC-8A: Instruction Manual. Browning. Retrieved from https://browningtrailcameras.zendesk.com/hc/en-us/article_attachments/360073084653/Spec_Ops_Advantage_BTC-8A.pdf.

[CR6] Buechel SD, Boussard A, Kotrschal A, van der Bijl W, Kolm N (2017) Brain size affects performance in a reversal-learning test. Proc R Soc B: Biol Sci 285(1871):20172031. 10.1098/rspb.2017.2031 10.1098/rspb.2017.2031 PMC5805926 29367391

[CR7] Carlson K, Gadziola M, Dauster E, Wesson D (2018) Selective attention controls olfactory decisions and the neural encoding of odors. Curr Biol 28(14):2195–2205 30056854 10.1016/j.cub.2018.05.011 PMC6530575

[CR8] Commodari E (2017) Novice readers: The role of focused, selective, distributed and alternating attention at the first year of the academic curriculum. I-Perception 8(4):204166951771855. 10.1177/2041669517718557 10.1177/2041669517718557 PMC5528191 28835811

[CR9] Cronk NE, Pillay N (2018) Food choice and feeding on carrion in two African mongoose species in an urban environment. Acta Ethol 21(2):127–136

[CR10] Cronk NE, Pillay N (2019a) Flexible use of urban resources by the yellow mongoose *Cynictis penicillata*. Animals 9(7):447. 10.3390/ani9070447 31315216 10.3390/ani9070447 PMC6680935

[CR11] Cronk N, Pillay N (2019b) Dietary overlap of two sympatric African mongoose species in an urban environment. Mammalia 83(5):428–438. 10.1515/mammalia-2018-0113

[CR12] Cronk NE, Pillay N (2020) Spatiotemporal co-occurrence and overlap of two sympatric mongoose species in an urban environment. J Urban Ecol 6(1):1–9. 10.1093/jue/juaa013

[CR13] Cronk NE, Pillay N (2021) Home range and use of residential gardens by yellow mongoose *Cynictis penicillata* in an urban environment. Urban Ecosyst 24(1):127–139. 10.1007/s11252-020-01022-1

[CR14] Croston R, Branch CL, Pitera AM, Kozlovsky DY, Bridge ES, Parchman TL, Pravosudov VV (2016) Predictably harsh environment is associated with reduced cognitive flexibility in wild food-caching mountain chickadees. Anim Behav 123:139–149. 10.1016/j.anbehav.2016.10.004

[CR15] Diamond A (2013) Executive functions. Ann Rev Psychol 64(1):135–168. 10.1146/annurev-psych-113011-143750 23020641 10.1146/annurev-psych-113011-143750 PMC4084861

[CR16] Dukas R, Kamil AC (2000) The cost of limited attention in blue jays. Behav Ecol 11(5):502–506. 10.1093/beheco/11.5.502

[CR17] Dukas R, Kamil AC (2001) Limited attention: The constraint underlying search image. Behav Ecol 12(2):192–199. 10.1093/beheco/12.2.192

[CR18] Federspiel IG, Garland A, Guez D, Bugnyar T, Healy SD, Güntürkün O, Griffin AS (2017) Adjusting foraging strategies: A comparison of rural and urban common mynas (*Acridotheres tristis*). Anim Cogn 20(1):65–74. 10.1007/s10071-016-1045-7 27778195 10.1007/s10071-016-1045-7

[CR19] Freas CA, LaDage LD, Roth TC, Pravosudov VV (2012) Elevation-related differences in memory and the hippocampus in mountain chickadees *Poecile gambeli*. Anim Behav 84(1):121–127. 10.1016/j.anbehav.2012.04.018

[CR20] Gill JA (2007) Approaches to measuring the effects of human disturbance on birds: Measuring the effects of human disturbance on birds. Ibis 149:9–14. 10.1111/j.1474-919X.2007.00642.x

[CR21] Ginsburg S, Jablonka E (2010) The evolution of associative learning: A factor in the Cambrian explosion. J Theor Biol 266(1):11–20. 10.1016/j.jtbi.2010.06.017 20558182 10.1016/j.jtbi.2010.06.017

[CR22] Gonzalez RC, Behrend ER, Bitterman ME (1967) Reversal learning and forgetting in bird and fish. Science 158(3800):519–521. 10.1126/science.158.3800.519 6069100 10.1126/science.158.3800.519

[CR23] Greggor AL, Clayton NS, Fulford AJC, Thornton A (2016) Street smart: Faster approach towards litter in urban areas by highly neophobic corvids and less fearful birds. Anim Behav 117:123–133. 10.1016/j.anbehav.2016.03.029 27429456 10.1016/j.anbehav.2016.03.029 PMC4938798

[CR24] Griffin AS, Guillette LM, Healy SD (2015) Cognition and personality: An analysis of an emerging field. Trends Ecol Evol 30(4):207–214. 10.1016/j.tree.2015.01.012 25736691 10.1016/j.tree.2015.01.012

[CR25] Griffiths SW, Brockmark S, Höjesjö J, Johnsson JI (2004) Coping with divided attention: The advantage of familiarity. Proc R Soc London Series B Biol Sci 271(1540):695–699. 10.1098/rspb.2003.2648 10.1098/rspb.2003.2648 PMC1691656 15209102

[CR26] Hahn B, Wolkenberg FA, Ross TJ, Myers CS, Heishman SJ, Stein DJ, Stein EA (2008) Divided versus selective attention: Evidence for common processing mechanisms. Brain Res 1215:137–146. 10.1016/j.brainres.2008.03.058 18479670 10.1016/j.brainres.2008.03.058 PMC2497334

[CR27] Izquierdo A, Brigman JL, Radke AK, Rudebeck PH, Holmes A (2017) The neural basis of reversal learning: An updated perspective. Neuroscience 345:12–26. 10.1016/j.neuroscience.2016.03.021 26979052 10.1016/j.neuroscience.2016.03.021 PMC5018909

[CR28] Jarjour C, Evans JC, Routh M, Morand-Ferron J (2019) Does city life reduce neophobia? A study on wild black-capped chickadees. Behav Ecol. 10.1093/beheco/arz167

[CR29] Kingdon J, Happold D, Butynski T, Hoffman M, Happold M, Kalina J (Eds.) (2013) *Cynictis penicillata*, Yellow Mongoose. Mammals of Africa. Vol. 5 Carnivores, pangolins, equids and rhinoceroses. Bloomsbury, London. pp. 334–339

[CR30] Klanker M, Feenstra M, Denys D (2013) Dopaminergic control of cognitive flexibility in humans and animals. Front Neurosci. 10.3389/fnins.2013.00201 24204329 10.3389/fnins.2013.00201 PMC3817373

[CR31] le Roux A, Cherry MI, Manser MB (2008) The audience effect in a facultatively social mammal, the yellow mongoose,* Cynictis penicillata*. Anim Behav 75(3):943–949. 10.1016/j.anbehav.2007.07.014

[CR32] Leal M, Powell BJ (2012) Behavioural flexibility and problem-solving in a tropical lizard. Biol Lett 8(1):28–30. 10.1098/rsbl.2011.0480 21752816 10.1098/rsbl.2011.0480 PMC3259950

[CR33] Lefebvre L, Reader SM, Sol D (2004) Brains, innovations and evolution in birds and primates. Brain, Behav Evol 63(4):233–246. 10.1159/000076784 15084816 10.1159/000076784

[CR34] Liu Y, Day LB, Summers K, Burmeister SS (2016) Learning to learn: Advanced behavioural flexibility in a poison frog. Anim Behav 111:167–172. 10.1016/j.anbehav.2015.10.018

[CR35] Lucon-Xiccato T, Bisazza A (2014) Discrimination reversal learning reveals greater female behavioural flexibility in guppies. Biol Lett 10(6):20140206. 10.1098/rsbl.2014.0206

[CR36] Mackintosh NJ, Mcgonigle B, Holgate V (1968) Factors underlying improvement in serial reversal learning. Can J Psychol/Revue Can de Psychol 22(2):85–95. 10.1037/h0082753 10.1037/h0082753 5649040

[CR37] Manser MB, Jansen DAWAM, Graw B, Hollén LI, Bousquet CAH, Furrer RD, le Roux A (2014) Vocal complexity in meerkats and other mongoose species. Adv Study Behav 46:281–310. 10.1016/B978-0-12-800286-5.00006-7

[CR38] Mazza V, Eccard JA, Zaccaroni M, Jacob J, Dammhahn M (2018) The fast and the flexible: Cognitive style drives individual variation in cognition in a small mammal. Anim Behav 137:119–132. 10.1016/j.anbehav.2018.01.011

[CR39] McLeod P (1977) A dual task response modality effect: Support for multiprocessor models of attention. Quart J Exper Psychol 29(4):651–667. 10.1080/14640747708400639

[CR40] Mettke-Hofmann C (2014) Cognitive ecology: Ecological factors, life-styles, and cognition: Cognitive ecology. Wiley Interdiscip Rev Cogn Sci 5(3):345–360. 10.1002/wcs.1289 26308568 10.1002/wcs.1289

[CR41] Müller M, Pillay N (2023) Learning and innovation in urban yellow mongooses (*Cynictis penicillata*). Ethology 129(11):600–611. 10.1111/eth.13396

[CR42] Nowak M, Sigmund K (1993) A strategy of win-stay, lose-shift that outperforms tit-for-tat in the Prisoner’s Dilemma game. Nature 364(6432):56–58. 10.1038/364056a0 8316296 10.1038/364056a0

[CR43] Papini MR (2002) Pattern and process in the evolution of learning. Psychol Rev 109(1):186–201. 10.1037/0033-295X.109.1.186 11863037 10.1037/0033-295x.109.1.186

[CR44] Parasuraman R (ed) (1998) The attentive brain: Issues and prospects. The Attentive Brain. Mass: MIT Press, Cambridge

[CR45] Parker MO, Gaviria J, Haigh A, Millington ME, Brown VJ, Combe FJ, Brennan CH (2012) Discrimination reversal and attentional sets in zebrafish (*Danio rerio*). Behav Brain Res 232(1):264–268. 10.1016/j.bbr.2012.04.035 22561034 10.1016/j.bbr.2012.04.035 PMC4167590

[CR46] R Core Team. (2013). R: A language and environment for statistical computing. R Foundation for Statistical Computing, Vienna, Austria. http://www.R-project.org/

[CR47] Rochais C, Hotte H, Pillay N (2021) Seasonal variation in reversal learning reveals greater female cognitive flexibility in African striped mice. Sci Rep 11(1):20061. 10.1038/s41598-021-99619-9 34625648 10.1038/s41598-021-99619-9 PMC8501043

[CR48] Do Linh San E., Cavallini P, Taylor P. (2015). The IUCN Red List of Threatened Species 2015 [Data set]. Int Union Conserv Nat. 10.2305/IUCN.UK.2015-4.RLTS.T41597A45205726.en

[CR49] Scott WA (1962) Cognitive Complexity and Cognitive Flexibility. Sociometry 25(4):405. 10.2307/2785779

[CR50] Sol D (2009) Revisiting the cognitive buffer hypothesis for the evolution of large brains. Biol Lett 5(1):130–133. 10.1098/rsbl.2008.0621 19049952 10.1098/rsbl.2008.0621 PMC2657766

[CR51] Sol D, Lapiedra O, González-Lagos C (2013) Behavioural adjustments for a life in the city. Anim Behav 85:1101–1112

[CR52] Sol D, Sayol F, Ducatez S, Lefebvre L (2016) The life-history basis of behavioural innovations. Philos Trans R Soc B Biol Sci 371(1690):20150187. 10.1098/rstb.2015.0187 10.1098/rstb.2015.0187 PMC4780529 26926277

[CR53] Spelke E, Hirst W, Neisser U (1976) Skills of divided attention. Cognition 4(3):215–230. 10.1016/0010-0277(76)90018-4

[CR54] St Clair JJH, García-Peña GE, Woods RW, Székely T (2010) Presence of mammalian predators decreases tolerance to human disturbance in a breeding shorebird. Behav Ecol 21(6):1285–1292. 10.1093/beheco/arq144

[CR55] Stanton LA, Bridge ES, Huizinga J, Johnson SR, Young JK, Benson-Amram S (2021) Variation in reversal learning by three generalist mesocarnivores. Anim Cogn 24(3):555–568. 10.1007/s10071-020-01438-4 33231749 10.1007/s10071-020-01438-4

[CR56] Sullivan L (1976) Selective attention and secondary message analysis: A reconsideration of Broadbent’s filter model of selective attention. Quart J Exper Psychol 28(2):167–178. 10.1080/14640747608400549

[CR57] Tebbich S, Teschke I (2014) Coping with uncertainty: Woodpecker finches (*Cactospiza pallida*) from an unpredictable habitat are more flexible than birds from a stable habitat. PloS One 9(3):e91718. 10.1371/journal.pone.0091718 24638107 10.1371/journal.pone.0091718 PMC3956741

[CR58] Tello-Ramos MC, Branch CL, Pitera AM, Kozlovsky DY, Bridge ES, Pravosudov VV (2018) Memory in wild mountain chickadees from different elevations: Comparing first-year birds with older survivors. Anim Behav 137:149–160. 10.1016/j.anbehav.2017.12.019

[CR59] Tello-Ramos MC, Branch CL, Kozlovsky DY, Pitera AM, Pravosudov VV (2019) Spatial memory and cognitive flexibility trade-offs: To be or not to be flexible, that is the question. Anim Behav 147:129–136. 10.1016/j.anbehav.2018.02.019

[CR60] Zentall TR (2005) Selective and divided attention in animals. Behav Process 69(1):1–15. 10.1016/j.beproc.2005.01.004 10.1016/j.beproc.2005.01.004 15795066

