# Supplementary material for: Cognitive flexibility in urban yellow mongooses, Cynictis penicillata
Source: Anim Cogn. 2024 Mar 2;27(1):14. doi: 10.1007/s10071-024-01839-9 (PMC10907452; doi:10.1007/s10071-024-01839-9)
Supplement: Supplementary file 1 — Supplementary file1 (PDF 1135 KB) [file 10071_2024_1839_MOESM1_ESM.pdf]

This file contains the Supplementary Information for the article titled:

**Cognitive flexibility in urban yellow mongooses, *Cynictis penicillata*.**

**Authors:** Mijke Müller, Neville Pillay.

School of Animal, Plant and Environmental Sciences. University of the Witwatersrand, Johannesburg, South Africa.

**Author for correspondence:** Mijke Müller (mullermijke@gmail.com)

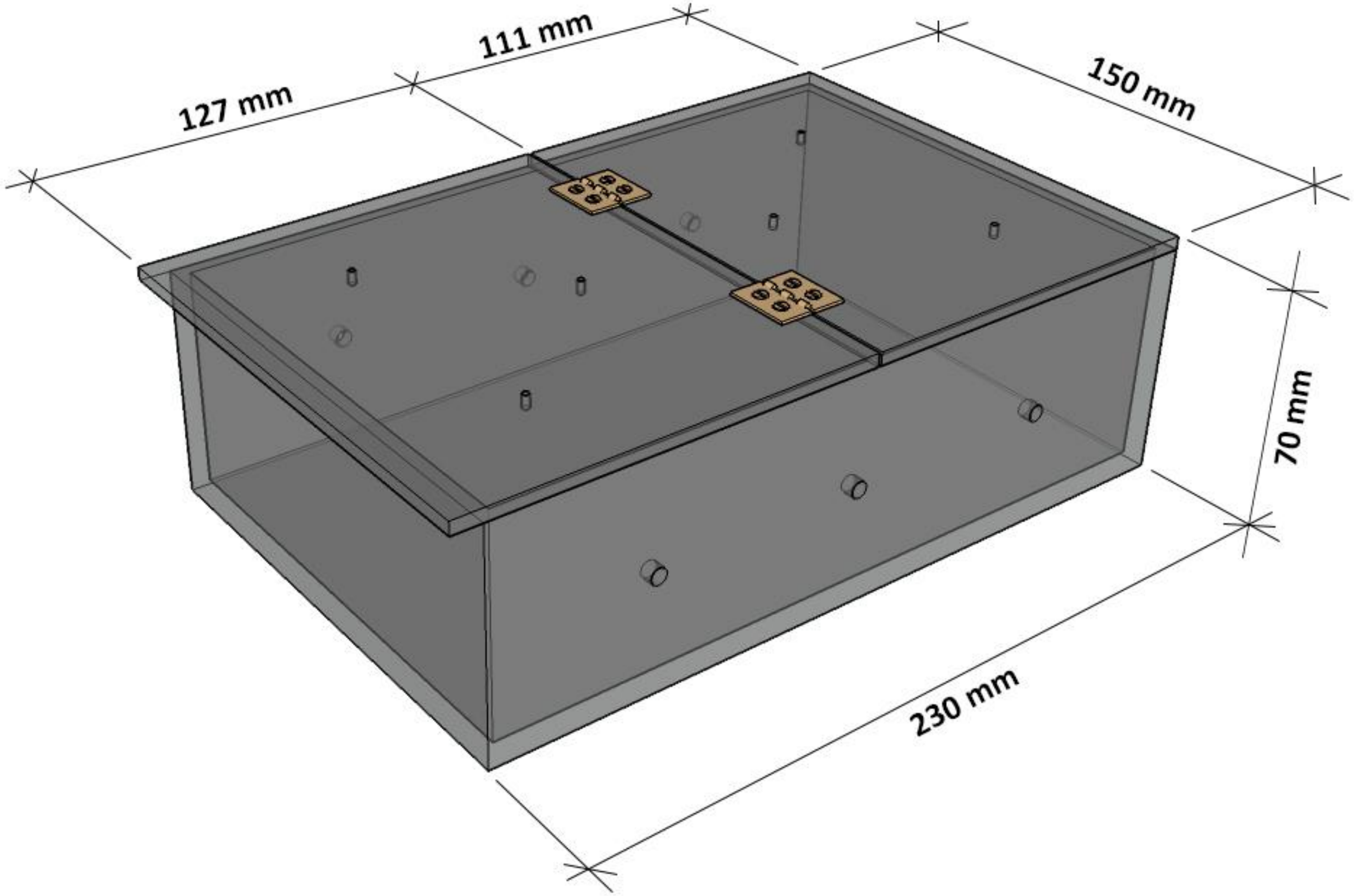


**Figure S1**. A diagram of a puzzle box used in this study. The puzzle boxes were made of transparent Perspex, allowing mongoose to detect food incentives visually. Small holes were present in the lid and sides of the box to allow mongoose to detect food incentives by smell. The boxes could be opened or closed because of two metal hinges.

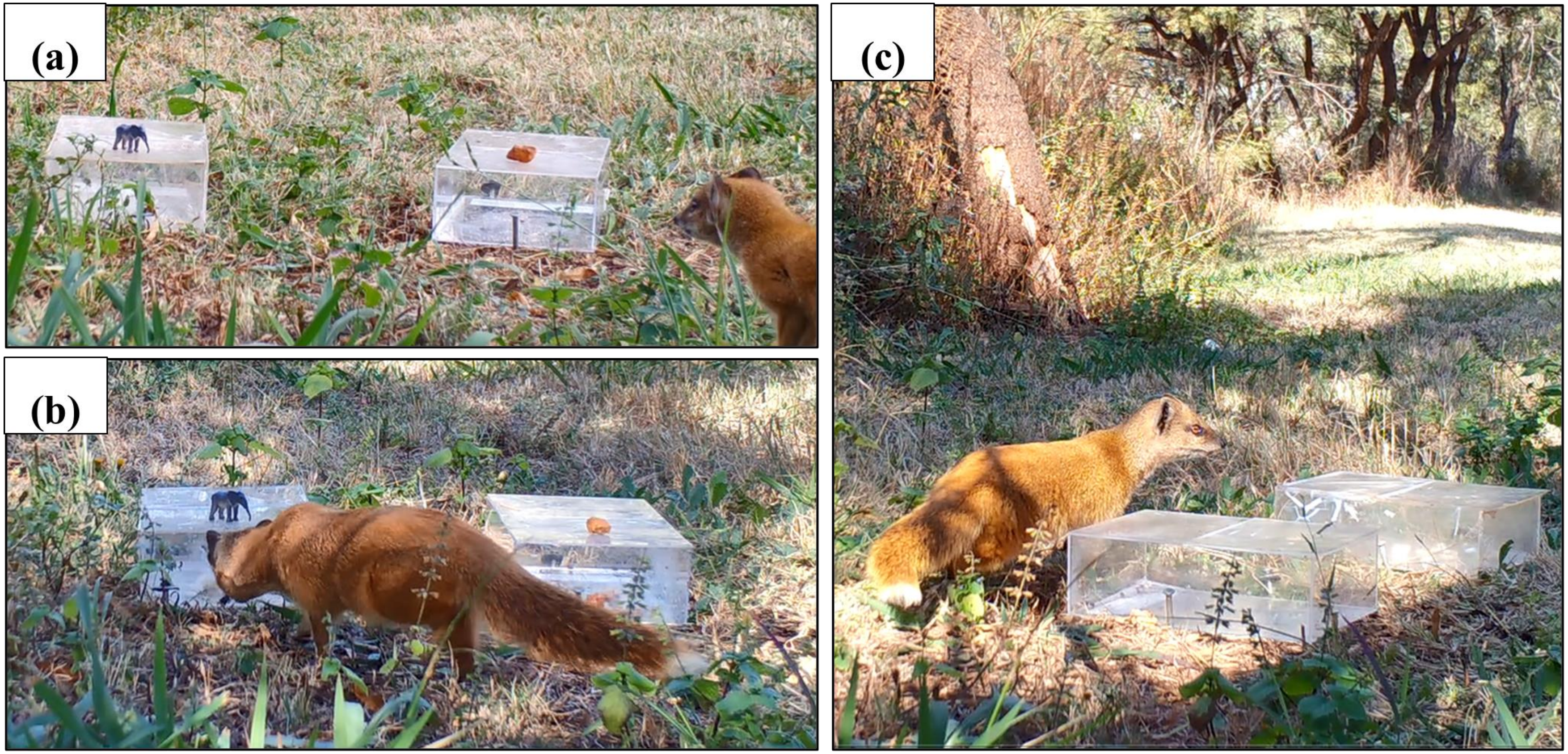


**Figure S2.** The experimental set-up of a reversal learning experiment conducted on yellow mongooses. **(a)** An associative learning phase where the left-most box contained the preferred food type (meat) with a small animal figurine on the lid of the box providing a visual cue, and the right-most box contained the non-preferred food type (bread) with a small pebble on the lid of the box providing a visual cue. **(b)** Reversal learning phase where the preferred and non-preferred food items were reversed so that each box contained the opposite food item (bread left and meat right). **(c)** A control test where the orientation of the boxes was changed, the objects removed from the lids, and the placement of the two food items randomised.

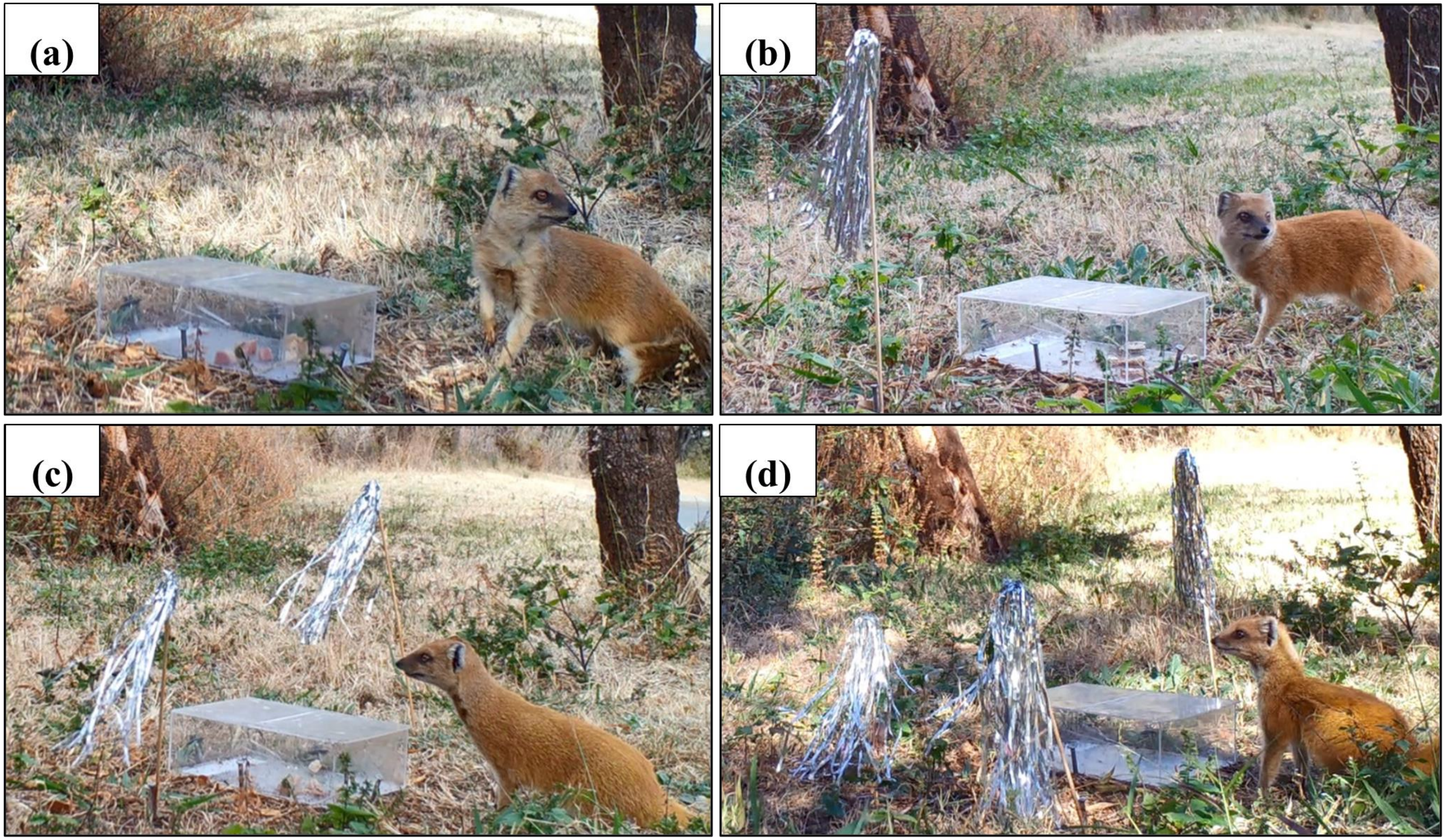


**Figure S3.** The experimental set-up of an attention task experiment conducted on yellow mongooses with **(a)** zero, **(b)** one, **(c)** two, and **(d)** three distractions surrounding the puzzle box. The number of distractions surrounding the puzzle box and the position of each distraction were randomised with each trial.
